# Geo-statistical analysis of *Culicoides* spp*.* distribution and abundance in Sicily, Italy

**DOI:** 10.1186/s13071-018-2658-2

**Published:** 2018-02-01

**Authors:** Valeria Blanda, Marcellocalogero Blanda, Francesco La Russa, Rossella Scimeca, Salvatore Scimeca, Rosalia D’Agostino, Michelangelo Auteri, Alessandra Torina

**Affiliations:** Istituto Zooprofilattico Sperimentale della Sicilia “A. Mirri”, Via Gino Marinuzzi, 3, 90129 Palermo, Italy

**Keywords:** *Culicoides*, *C. imicola*, *C. obsoletus* complex, *C. pulicaris*, Abundance, GIS, Inverse distance weighted method, Sicily, Italy

## Abstract

**Background:**

Biting midges belonging to *Culicoides imicola*, *Culicoides obsoletus* complex and *Culicoides pulicaris* complex (Diptera: Ceratopogonidae) are increasingly implicated as vectors of bluetongue virus in Palaearctic regions. *Culicoides obsoletus* complex includes *C. obsoletus* (*sensu stricto*), *C. scoticus*, *C. dewulfi* and *C. chiopterus*. *Culicoides pulicaris* and *C. lupicaris* belong to the *Culicoides pulicaris* complex. The aim of this study was a geo-statistical analysis of the abundance and spatial distribution of *Culicoides* spp. involved in bluetongue virus transmission. As part of the national bluetongue surveillance plan 7081 catches were collected in 897 Sicilian farms from 2000 to 2013.

**Methods:**

Onderstepoort-type blacklight traps were used for sample collection and each catch was analysed for the presence of *Culicoides* spp. and for the presence and abundance of *Culicoides* vector species (*C. imicola*, *C. pulicaris / C. obsoletus* complexes). A geo-statistical analysis was carried out monthly *via* the interpolation of measured values based on the Inverse Distance Weighted method, using a GIS tool. Raster maps were reclassified into seven classes according to the presence and abundance of *Culicoides*, in order to obtain suitable maps for Map Algebra operations.

**Results:**

Sicilian provinces showing a very high abundance of *Culicoides* vector species were Messina (80% of the whole area), Palermo (20%) and Catania (12%). A total of 5654 farms fell within the very high risk area for bluetongue (21% of the 26,676 farms active in Sicily); of these, 3483 farms were in Messina, 1567 in Palermo and 604 in Catania. *Culicoides imicola* was prevalent in Palermo, *C. pulicaris* in Messina and *C. obsoletus* complex was very abundant over the whole island with the highest abundance value in Messina.

**Conclusions:**

Our study reports the results of a geo-statistical analysis concerning the abundance and spatial distribution of *Culicoides* spp*.* in Sicily throughout the fourteen year study. It provides useful decision support in the field of epidemiology, allowing the identification of areas to be monitored as bases for improved surveillance plans. Moreover, this knowledge can become a tool for the evaluation of virus transmission risks, especially if related to vector competence.

## Background

*Culicoides* biting midges (Diptera: Ceratopogonidae) are small hematophagous insects widely spread through different geographical areas, from America to Europe, Asia and Oceania [1]. Breeding sites for *Culicoides* include a wide range of habitats often located nearby their hosts, in and around farm holdings, such as decaying vegetation, dung, pond borders and moist soils. Furthermore, their larvae can survive within aquatic sites characterized by different ranges of acidity and salinity [2]. Blood-feeding is required for egg production by females, which bite hosts such as amphibians, birds and mammals including humans and domestic animals [3].

Different species included in the genus *Culicoides* are of veterinary importance as vectors of different arboviruses causing severe animal diseases. Bluetongue virus (BTV) and African horse sickness virus (AHSV), listed as causative agents of globally important diseases by the OIE (Office International des Epizooties), are transmitted by *Culicoides* species [3]. Moreover, the species of the *Culicoides obsoletus* complex have been recently considered the potential vectors of the Schmallenberg virus (SVB), a virus that was not previously detected in Europe [4]. *Culicoides* spp. can also transmit filarial diseases such as onchocercosis and mansonellosis, affecting various species including humans [3, 5].

Concerning BTV infection, just about 30 *Culicoides* species have fulfilled the criteria required to be considered as BTV vectors, due to a series of barrier systems preventing virus replication within the biting midges. Recognized BTV vectors include *C. imicola* (the main vector in Africa, the Middle East, Southeast Asia and areas of southern Europe), *C. sonorensis* (the main vector in North America) and *C. brevitarsis* (the main vector in Australia) [6]. Wind-borne transportation of *Culicoides* or accidental importation of infected hosts can contribute to BTV entry in novel areas [7]. Spreading of species such as *Culicoides imicola* was possibly favoured by ongoing climate changes, allowing its diffusion in northern Europe as well as an increase of the seasonal activity period, vector density and virus infections susceptibility [8].

In Europe, BTV vectors belong to three main species or species complexes [9]. Species within the *Culicoides obsoletus* complex, i.e. *Culicoides obsoletus* (*sensu stricto*), *Culicoides scoticus*, *Culicoides dewulfi* and *Culicoides chiopterus*, are putative BTV vectors in northern and central Europe [10, 11]. Other relevant BTV vector species belong to the *Culicoides pulicaris* complex [12]. Finally, *Culicoides imicola* is the most important vector in the Mediterranean basin and it may have a role in northern Europe [12]. Within the Mediterrean, Sicily (Italy) has geographical and climatic features particularly suitable for introduction and spread of *Culicoides* vectors. Sicily has a typical Mediterranean climate, with mild winters, warm autumns/springs and hot summers; however, temperatures can vary among areas depending on the distance from the sea and the presence of mountains.

Studies on the abundance and distribution of *Culicoides* species involved in vector-borne diseases are essential to define infection risk areas, as well as to identify possible local factors favouring the diffusion of *Culicoides* and associated pathogens. For such purposes, geographical information system (GIS) technologies have become useful tools for disease mapping, ecological analyses, prediction of parasite occurrence/seasonality and surveillance of parasitic diseases [13]. Thus, GIS technology is increasingly used to carry out a systematic analysis of spatial distribution of vectors and related diseases, usable for the development of effective countermeasures against arthropod-borne diseases.

Our study was focused on a geo-statistical analysis of the abundance and spatial distribution of *Culicoides* involved in the bluetongue virus transmission, elaborating data obtained from a long monitoring period (2000–2013) in Sicily. Our results will be of importance for improving current surveillance plans in Sicily and may represent a basis for a systematic use of GIS in *Culicoides*-trasmitted disease risk analysis.

## Methods

### Vector monitoring

**Fig. 1:**
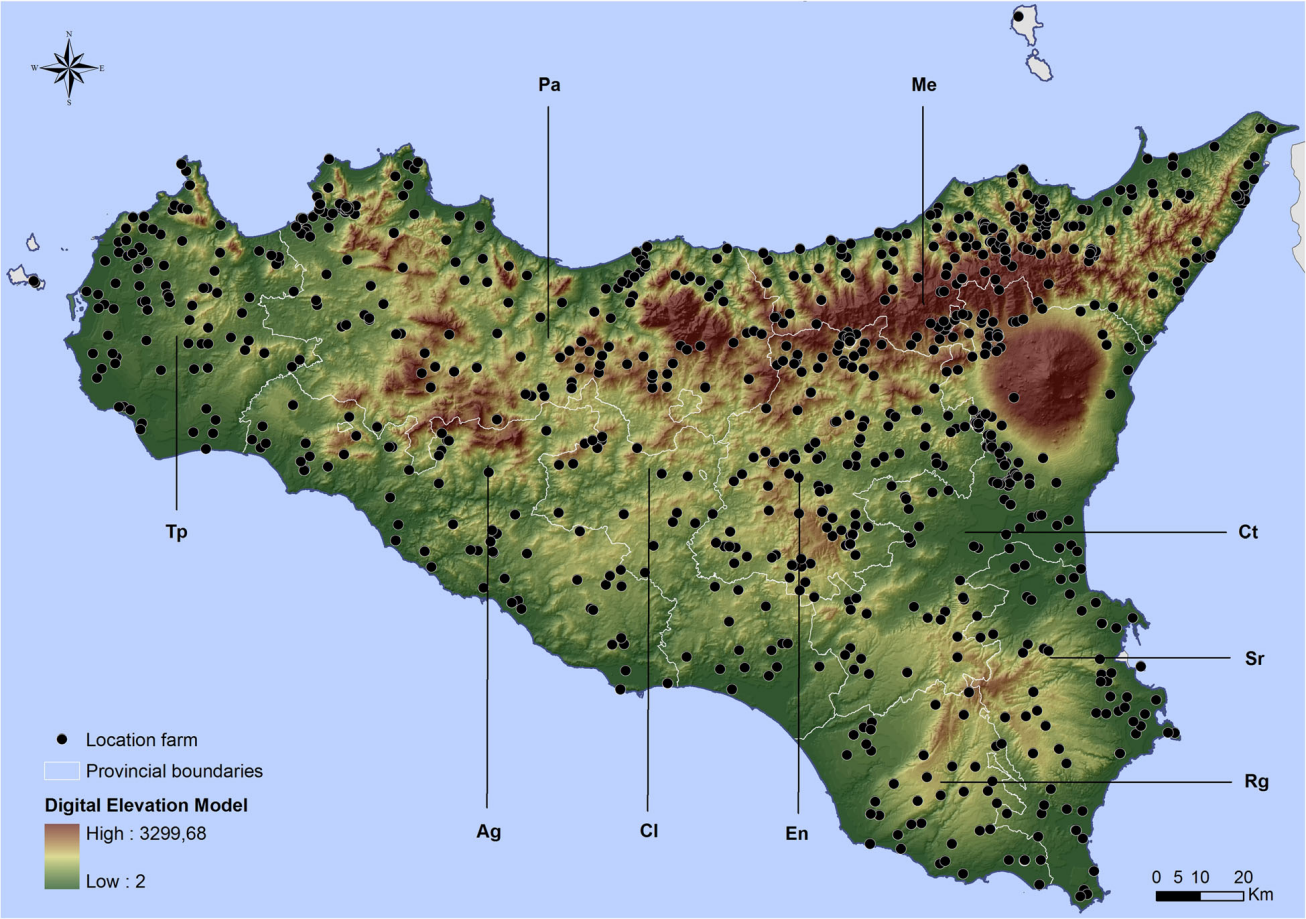
Location of farms. The farms were located using geographical coordinates measured on the field with GPS and reported on a digital elevation model. The digital elevation model was processed through the interpolation of level curve values of the Sicilian region

Farms included in our study were part of the National Entomologic Surveillance Program for Bluetongue under the supervision of the Italian Ministry of Health, and thus they were chosen in order to cover the entire territory of Sicily ensuring as much as possible a uniform distribution throughout the different provinces, as well as in consideration of ecological factors (e.g. altitude, distance from the sea, vegetation). From 2000 to 2013, 7081 catches have been collected in 897 farms distributed in Sicily (Fig. 1). Catches were collected in cattle, sheep and goat farms through the use of Onderstepoort-type blacklight suction traps [14]. In addition to mobile traps, stationary traps were also placed in each of the different Sicilian provinces (Agrigento, Caltanissetta, Catania, Enna, Messina, Palermo, Ragusa, Siracusa and Trapani). This standardized trap type is commonly used in monitoring and surveillance programs, since it is very efficient compared to other suction light traps [15]. Traps were hung at a height of 1.5 m above the ground; the distance from stables and paddocks with cattle and sheep was less than 20 m and traps were activated from sunset (07:00 pm) to sunrise (08:00 am). For each farm, traps were activated once a week (4 nights per month).

The insects, attracted by the UV light, were sucked into plastic containers containing 200–300 ml of water in which a few drops of detergent were added to reduce surface tension. Collected insects were then placed in vials filled with 70% ethanol.

*Culicoides* spp. were first separated from other insects under a stereomicroscope. Afterwards, *Culicoides* species/complexes were identified by the characteristic wing patterns and spots [16–18]. In particular, regarding the species most involved in pathogen transmission, *C. imicola* specimens were recorded from 2000 to 2013, while the occurrence of species belonging to the *C. obsoletus* and *C. pulicaris* complexes was analysed from 2005 to 2013.

### Spatial analysis

Results were recorded in a database and the monthly average abundance was calculated for each farm. The monthly average was chosen as a reliable parameter for estimating *Culicoides* abundance, since it may vary in the Sicilian territories throughout a month, in a negative or positive way due to transitory adverse or favourable weather conditions. For every month, a geo-statistical analysis *via* a GIS tool was performed by interpolating measured values with the IDW (inverse distance weighted) method, one of the most important tools in GIS-based spatial interpolation. The IDW method was used for data analysis considering its easy implementation and its suitability to the characteristics of our data, compared to other interpolation methods. This methodology allowed the estimation of values in unsampled points focusing on the basic assumption that values in unsampled points are the weighted average of values in nearby sampled points. Weights are elaborated as the inverse squares of distances. The final objective of IDW analysis is the creation of a smooth surface where values in unsampled points are more similar to nearby points than distant points [19].

In detail, 12 monthly maps were processed for each species. Once processed, each raster map was reclassified into 7 classes in relation to *Culicoides* presence and abundance (0, absent to 6, very high), giving a score for each class to each pixel of the image. The abundance classes were the same for each month and for each *Culicoides* species, and they were defined using the same threshold values.

**Fig. 2:**
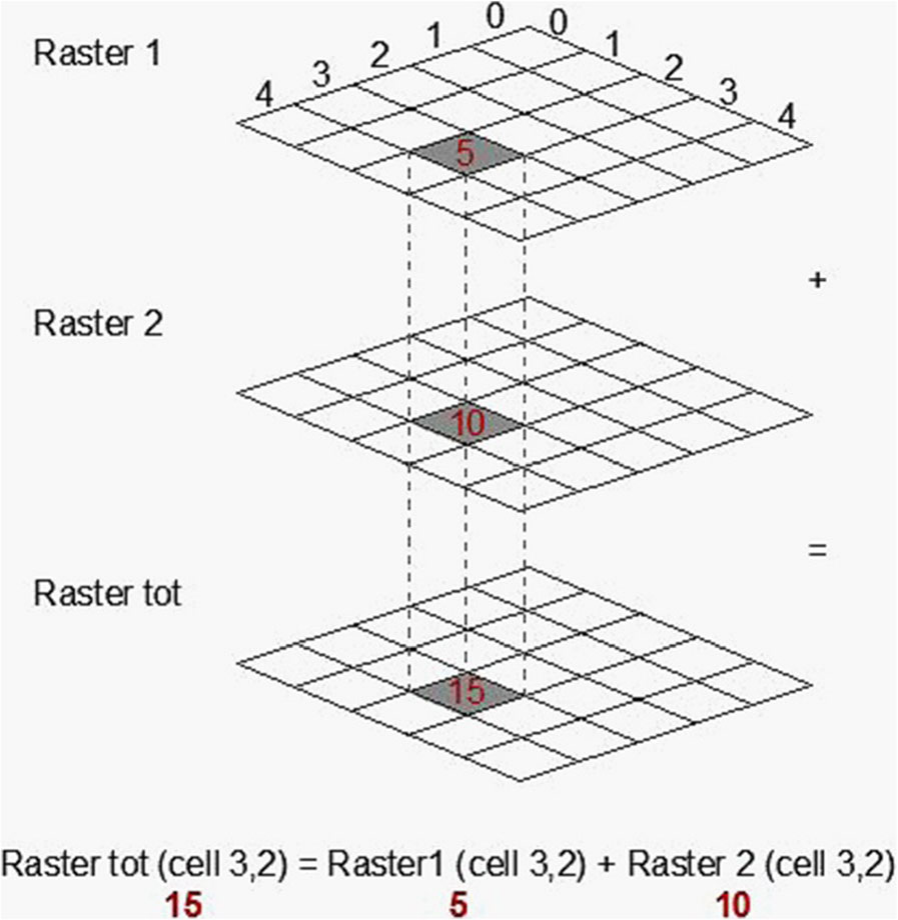
Algebra map (sum of raster). Using the algebra map, the sum ​​of the pixel values ​​from the reclassified raster images was used to develop distribution and abundance maps of *Culicoides* species

The 12 monthly maps were drawn from IDW maps for each of the three *Culicoides* species or complex of species considered as the most important bluetongue virus vectors. Areas with cells showing the highest values represent the ones with high levels of *Culicoides*. The reclassification allowed us to draw comparable maps that permit Map Algebra operations, such as summation (Fig. 2).

Through the sum of the monthly maps, it was possible to draw up a map of abundance for each of the three *Culicoides* species/complexes (*C. imicola*, *C. pulicaris* and *C. obsoletus*). Such maps were further processed to produce a general map comprehensive of all the three species, from which a final map reporting abundance classes in all the Sicilian provinces was elaborated with the evidence of municipalities at risk.

## Results

Results of the monitoring carried out from 2000 to 2013, with a particular attention to the *Culicoides* species/complexes most involved in the bluetongue virus transmission (*C. imicola, C. pulicaris* and *C. obsoletus*), are reported in Table 1. In addition, the following other *Culicoides* species were recorded: *C. agathensis*, *C. cataneii*, *C. circumscriptus*, *C. fagineus*, *C. fascipennis*, *C. festivipennis*, *C. gejgelensis*, *C. heteroclitus*, *C. kijng*, *C. monoculicoides*, *C. newsteadi*, *C. nubeculosis*, *C. paulae*, *C. pictipennis*, *C. punctatus*, *C. puncticollis*, *C. schultzei*, *C. stigma*, *C. subfascipennis* and *C. tauricus* (data not shown).

**Table 1:** Abundance of *Culicoides* spp., *C. imicola*, *C. obsoletus* complex and *C. pulicaris* complex based on 7081 catches during 2000–2013

	No. of specimens	Monitoring period
Insects	4,815,500	2000–2013
*Culicoides* spp.	954,776	2000–2013
*C. imicola*	78,278	2000–2013
*C. obsoletus* complex	265,575	2005–2013
*C. pulicaris* complex	9099	2005–2013

**Fig. 3:**
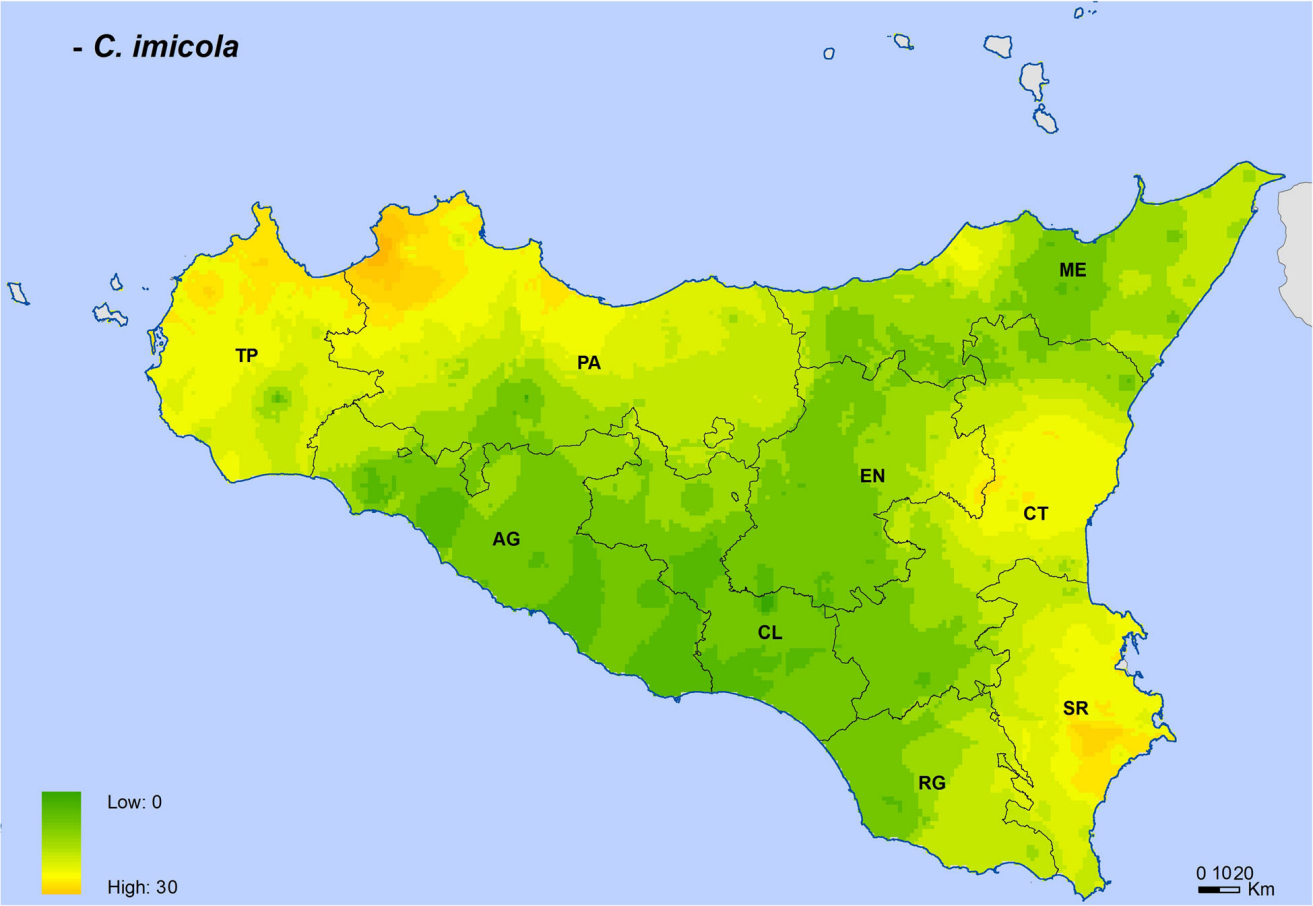
*Culicoides imicola* distribution and abundance map. The map was created using the Esri ArcMap 9.3 software. *Abbreviations*: AG, Agrigento; CL, Caltanissetta; CT, Catania; EN, Enna; ME, Messina; PA, Palermo; RG, Ragusa; SR, Siracusa; TP, Trapani

**Fig. 4:**
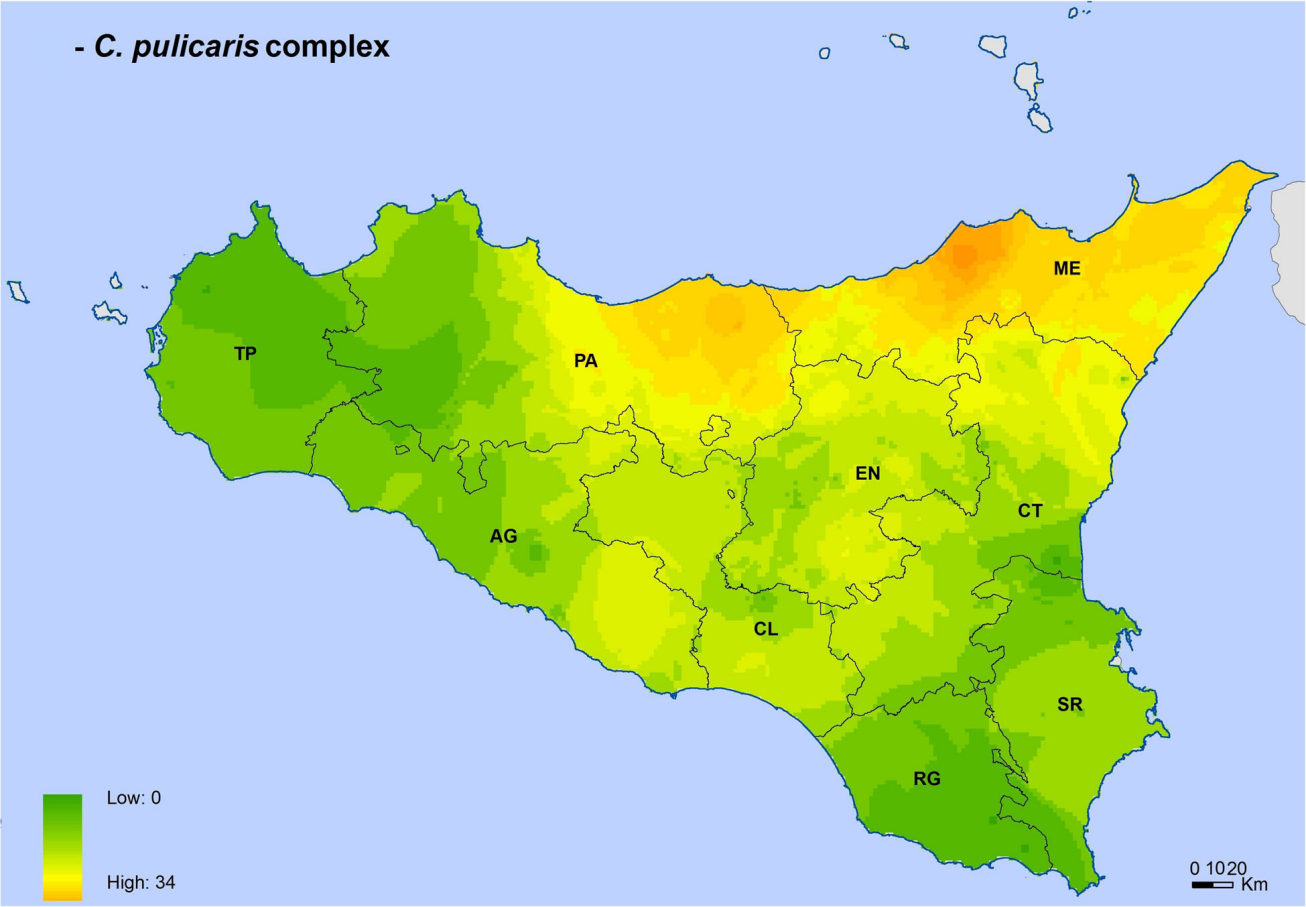
*Culicoides pulicaris* complex distribution and abundance map. The map was created using the Esri ArcMap 9.3 software. *Abbreviations*: AG, Agrigento; CL, Caltanissetta; CT, Catania; EN, Enna; ME, Messina; PA, Palermo; RG, Ragusa; SR, Siracusa; TP, Trapani

**Fig. 5:**
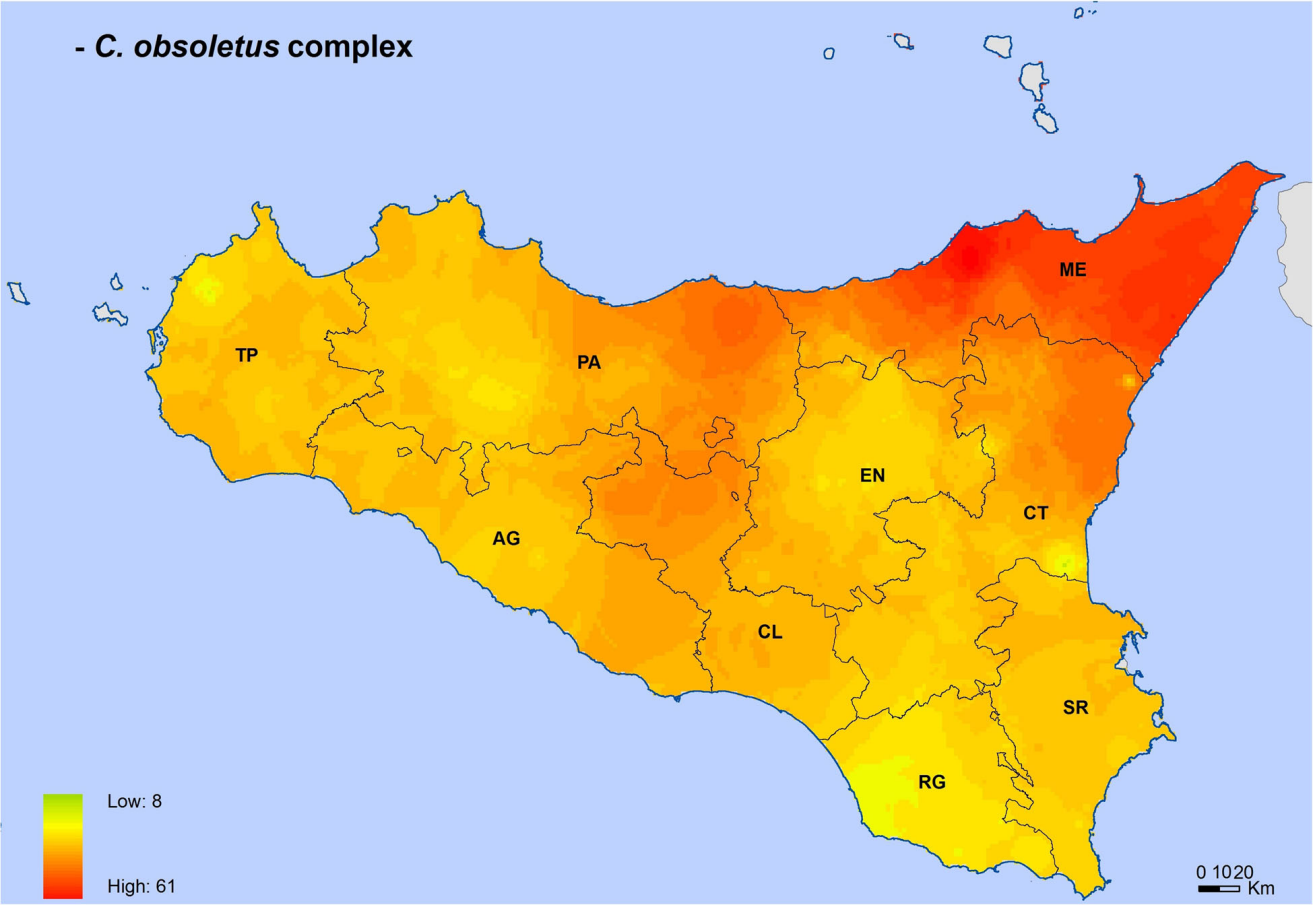
*Culicoides obsoletus* complex distribution and abundance map. The map was created using the Esri ArcMap 9.3 software. *Abbreviations*: AG, Agrigento; CL, Caltanissetta; CT, Catania; EN, Enna; ME, Messina; PA, Palermo; RG, Ragusa; SR, Siracusa; TP, Trapani

**Fig. 6:**
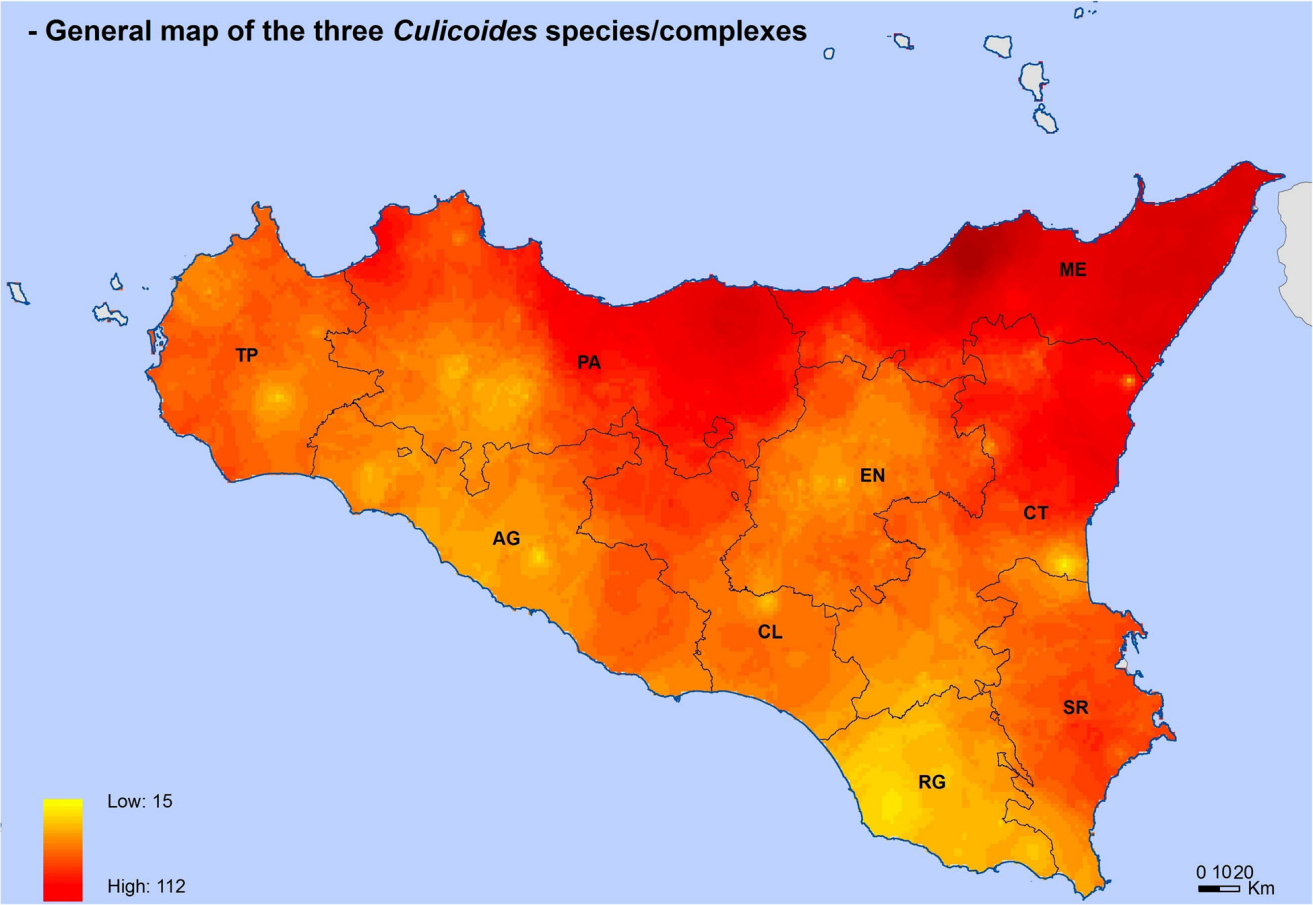
Distribution and abundance map of all the three *Culicoides* species/complexes. The map was created using the Esri ArcMap 9.3 software. *Abbreviations*: AG, Agrigento; CL, Caltanissetta; CT, Catania; EN, Enna; ME, Messina; PA, Palermo; RG, Ragusa; SR, Siracusa; TP, Trapani

Maps for *C. imicola*, *C. obsoletus* complex and *C. pulicaris* complex showed their different spatial distributions (Figs. 3-6). *Culicoides imicola* was widespread in Palermo province, followed by Siracusa and Trapani provinces. This species was less abundant in the southern part of the island, in Agrigento and Caltanissetta provinces (Fig. 3). *Culicoides pulicaris* complex was mainly found in Messina province and in the eastern area of Palermo province, whilst it was scarcely present in some areas of Trapani, Palermo and Ragusa provinces (Fig. 4). *Culicoides obsoletus* complex was especially abundant within the territory of Messina province, while it was less abundant in some areas of Trapani, Catania and Ragusa provinces (Fig. 5). The general map including the three *Culicoides* species/complexes showed that they were present in the whole of Sicily, with the highest levels in Messina province, followed by the eastern part of Palermo province and the northern area of Catania province. The lowest levels were found in Ragusa province (Fig. 6).

**Fig. 7:**
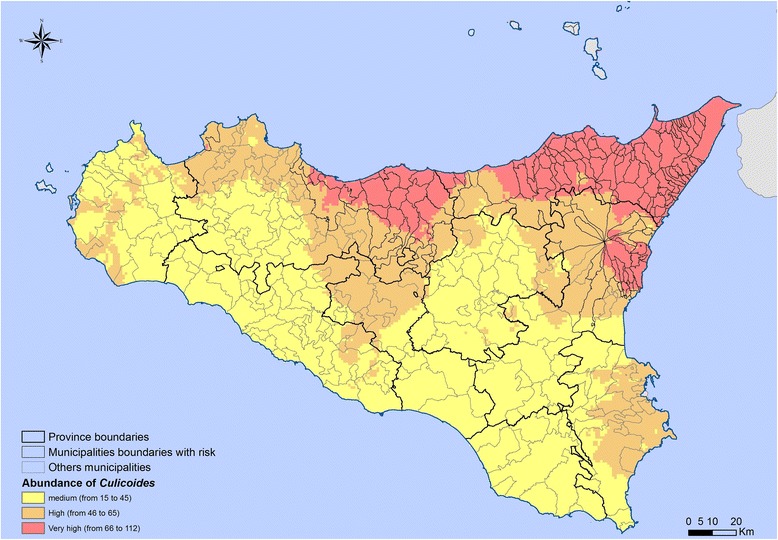
Abundance map of all the three *Culicoides* species/complexes with evidence of municipalities at risk. The map shows the areas and the municipalities with medium, high and very high *Culicoides* abundance and allows calculation of the percentage of the territories of each province. *Abbreviations*: AG, Agrigento; CL, Caltanissetta; CT, Catania; EN, Enna; ME, Messina; PA, Palermo; RG, Ragusa; SR, Siracusa; TP, Trapani

The final map reporting the abundance classes for the three species/complexes (Fig. 7) highlighted that the Sicilian provinces fell into three classes (medium, high and very high abundance). The percentage of land falling in the different classes for each province (Table 2) showed that provinces at very high-risk are Messina (80%), Palermo (20%) and Catania (12%), while a medium risk levels could be noticed in Ragusa. The number of farms (ovine and bovine) that fell within the very high risk area was 5654, corresponding to 21% of the 26,676 farms active in Sicily; of these, 3483 farms fell in Messina province, 1567 in Palermo province and 604 in Catania province.

**Table 2:** Percentages of areas with different *Culicoides* abundance in Sicilian provinces

Province	Medium	High	Very high
Trapani	76	24	–
Palermo	31	49	20
Messina	1	19	80
Agrigento	88	12	–
Caltanissetta	63	37	–
Enna	80	20	–
Catania	47	41	12
Ragusa	100	–	–
Siracusa	52	48	–

## Discussion

Our work reports the results of a geo-statistical approach applied to the study of the abundance of some relevant *Culicoides* vector species, analysing a significant number of catches distributed all over the seasons during 14 years. Our approach allows integration of the field data with the ones resulting from the GIS-based IDW method, permitting the elaboration of comprehensive maps including areas where no measurements were available.

As reported, *Culicoides* spp. are spread all over Sicilian territories, with *C. imicola* particularly abundant in Palermo, and *C. pulicaris* complex and *C. obsoletus* complex being particularly abundant in Messina. Considering all the three species together (Fig. 7), *Culicoides* are particularly abundant in Messina province and less abundant in Ragusa territories.

Several factors may influence the spread of *Culicoides* spp., including both biotic (e.g. vegetation, human presence, other animal presence) and abiotic (light, soil, water, air, climatic factors etc.) factors as well as other factors that can vary in terms of macrohabitat or microhabitat. All of these factors are implicated in producing substantial differences in the presence and abundance of *Culicoides* spp., and local variations occur also at short distance. As an example, *C. imicola,* is widely distributed across the world, from South Africa to the Mediterranean basin and the Middle and Far East [20]. There are, however, areas with either low abundance or even completely free of *C. imicola* [14], underlining a patchy distribution of this species. Other studies have confirmed that the spatial distribution of *Culicoides* spp. could be influenced both by different climatic zones [21] as well as by local factors, such as flock management systems. Influence of seasonal and metereological parameters on *Culicoides* activity was also investigated [22–25] and other studies reported risk maps obtained *via* the analysis of habitat characteristics [26–28]. As an example, an association between the bluetongue disease diffusion and some landscape metrics, such as woodland and open prairies, was revealed [29].

Accordingly, even in our study we observed an irregular distribution of *Culicoides* abundance in Sicily that could also be explained by a possible correlation to specific biotic or abiotic factors in the different provinces. For instance, the high number of farms falling in high risk territories in Messina province could be explained by the farm type, mostly represented by family-owned farms with a small number of animals. Conversely, in Catania province industrial farms with a great number of animals are common. Other factors could influence the distribution pattern of *Culicoides* in Sicily, including different vegetation, animal presence and microclimatic factors such as altitude, temperature and precipitation. In this study, we provided a picture of *Culicoides* presence in the Sicilian territories; we related the risk for *Culicoides*-transmitted diseases with the *Culicoides* abundance in the different provinces. However, several facets of the data can be further examined and developed, as indeed the correlation with environmental factors and seasonality, which would be our future perspectives of analysis. At present, we have provided an overview about the annual overall abundance of each species, reporting maps obtained by the sum of monthly maps. However, in the perspective of seasonality studies, the specific abundance values within each season/month could be used for correlation with seasonal/monthly environmental parameters. In this view, the implementation of modelling analysis would provide a useful tool to enhance data analysis and to uncover the multiform facets connecting entomological field data to environmental factors, population dynamics, and pathogen diffusion.

The significance of the maps we obtained should also be evaluated in relation to the vector competence of a species towards a pathogen. In Sicily, for example, the bluetongue outbreaks that occurred from 2000 to 2013 severely affected Trapani province, as well as the western part of Palermo province. These occurrences can be explained analysing the map of *C. imicola* that is a very competent vector of bluetongue virus (Fig. 3). Similarly, to evaluate the risk of Schmallenberg virus spreading, the map reporting *C. obsoletus* complex distribution has to be considered.

However, the transmission of the same pathogen may be due to several *Culicoides* species with different vector competence. Thus, correction factors relative to the competence of a *Culicoides* species towards the examined pathogen should be considered for map elaboration. However, studies on vector competence are complex, requiring highly specialized laboratories, pathogen availability and laboratory colonies of the vector. Nevertheless, for some vectors and pathogens, this information is available. For example, a study conducted on vector competence of *C. sonorensis* [30] showed that this species has a competence of 32% towards epizootic hemorragic disease virus and this information certainly allows a precise assessment of the disease risk correlated to this species. Studies providing novel data on competence for the other *Culicoides* species involved in pathogen transmission could lead to the elaboration of improved maps that better define the risk for *Culicoides*-related diseases.

## Conclusions

In conclusion, this study uses a GIS-based geo-statistical approach to perform a comprehensive analysis of abundance and spatial distribution of *Culicoides* spp. most involved in virus transmission. We reported data derived from a long time surveillance period (2000–2013). Our study confirmed the high abundance of *Culicoides* species in Sicily, underlining the presence of *C. imicola* in Palermo province and of *C. pulicaris* complex and *C. obsoletus* complex in Messina province. The analysis provides a useful tool for decision support in the field of epidemiology, allowing the identification of areas to focus on for surveillance purposes. Indeed, the data showed the presence of medium- to high-risk areas in the whole Sicily and of a particularly high-risk area in Messina province. Moreover, if related to vector competence, these data can become an instrument for the prevention of pathogen transmission risks.

## References

[1] Borkent A. World species of biting midges (Diptera: Ceratopogonidae), 2012. http://wwx.inhs.illinois.edu/files/8413/4219/9566/CeratopogonidaeCatalog.pdf. Accessed 17 May 2017.

[2] González M, López S, Mullens BA, Baldet T, Goldarazena A (2013). A survey of *Culicoides* developmental sites on a farm in northern Spain, with a brief review of immature habitats of European species. Vet Parasitol.

[3] Mellor PS, Boorman J, Baylis M. *Culicoides* biting midges: their role as arbovirus vectors. Annu Rev Entomol. 2000;45:307–40. 10.1146/annurev.ento.45.1.307 10761580

[4] Rasmussen LD, Kristensen B, Kirkeby C, Rasmussen TB, Belsham GJ, Bødker R, Bøtner A (2012). Culicoids as vectors of Schmallenberg virus. Emerg Infect Dis.

[5] Linley JR, Hoch AL, Pinheiro FP (1983). J Med Entomol.

[6] Wilson AJ, Mellor PS (2009). Bluetongue in Europe: past, present and future. Phil Trans R Soc B.

[7] Barros SC, Ramos F, Luis TM, Vaz A, Duarte M, Henriques M (2007). Molecular epidemiology of bluetongue virus in Portugal during 2004–2006 outbreak. Vet Microbiol.

[8] Purse BV, Mellor PS, Rogers DJ, Samuel AR, PPC M, Baylis M (2005). Climate change and the recent emergence of bluetongue in Europe. Nat Rev Microbiol.

[9] Wilson AJ, Mellor PS (2008). Bluetongue in Europe: vectors, epidemiology and climate change. Parasitol Res.

[10] Meiswinkel R, Baldet T, De Deken R, Takken W, Delecolle JC, Mellor PS (2008). The 2006 outbreak of bluetongue in northern Europe - the entomological perspective. Prev Vet Med..

[11] De Liberato C, Scavia G, Lorenzetti R, Scaramozzino P, Amaddeo D, Cardeti G, et al. Identification of *Culicoides obsoletus* (Diptera: Ceratopogonidae) as a vector of bluetongue virus in central Italy. Vet Rec. 2005;156:301–4. 10.1136/vr.156.10.301 15786918

[12] Caracappa S, Torina A, Guercio A, Vitale F, Calabro A, Purpari G, et al. Identification of a novel bluetongue virus vector species of *Culicoides* in Sicily. Vet Rec. 2003;153:71–4. 10.1136/vr.153.3.71 12892265

[13] Cringoli G, Rinaldi L, Veneziano V, Musella V (2005). Disease mapping and risk assessment in veterinary parasitology: some case studies. Parassitologia.

[14] Venter GJ, Meiswinkel R (1994). The virtual absence of *Culicoides imicola* (Diptera: Ceratopogonidae) in a light-trap survey of the colder, high-lying area of the eastern Orange free state, South Africa, and implications for the transmission of arboviruses. Onderstepoort J Vet Res.

[15] Venter GJ, Labushagne K, Hermanides KG, SNB B, Majatladi DM (2009). Comparison of the efficiency of five suction light traps underfield condition in South Africa for the collection of *Culicodes* species. Vet Parasitol.

[16] Goffredo M, Meiswinkel R (2004). Entormological Surveillance of bluetongue in Italy: method of capture, catch analysis and identification of *Culicoides* biting midges. Vet Ital.

[17] Delécolle JC. Nouvelle contribution a l’etude systematique et iconographique des especes du genre Culicoides (Diptera: Ceratopogonidae) du Nord-Est de la France. Strasbourg: Universite Louis Pasteur de Strasbourg, “Vie et Terre”; 1985.

[18] Rawlings P (1996). A key, based on wing patterns of biting midges (genus: *Culicoides* Latreille - Diptera: Ceratopogonidae) in the Iberian peninsula, for use in epidemiological studies. Graellsia.

[19] Longley PA, Goodchild MF, Maguire DJ, Rhind DW (2005). Geographic information system and science.

[20] Meiswinkel R, Nevill EM, Venter GJ, Coetzer JAW, Thomson GR, Tustin RC (1994). Vectors: *Culicoides* spp. Infectious diseases of livestock with special reference to southern Africa.

[21] Brugger K, Rubel F (2013). Characterizing the species composition of European *Culicoides* vectors by means of the Köppen-Geiger climate classification. Parasit Vectors.

[22] Brugger K, Rubel F (2013). Bluetongue disease risk assessment based on observed and projected *Culicoides obsoletus* spp. vector densities. PLoS One.

[23] Sanders CJ, Shortall CR, Gubbins S, Burgin L, Gloster J, Harrington R, et al. Influence of season and meteorological parameters on flight activity of *Culicoides* biting midges. J Appl Ecol. 2011;48:1355–136.

[24] Racloz V, Venter G, Griot C, KDC S (2008). Estimating the temporal and spatial risk of bluetongue related to the incursion of infected vectors into Switzerland. BMC Vet Res.

[25] Ander M, Meiswinkel R, Chirico J (2012). Seasonal dynamics of biting midges (Diptera: Ceratopogonidae: *Culicoides*), the potential vectors of bluetongue virus, in Sweden. Vet Parasitol.

[26] Conte A, Ippoliti C, Calistri P, Pelini S, Savini L, Salini R, et al. Towards the identification of potential infectious sites for bluetongue in Italy: a spatial analysis approach based on the distribution of *Culicoides imicola*. Vet Ital. 2004;40:311–5. 20419684

[27] Takken W, Verhulst N, Scholte EJ, Jacobs F, Jongema Y, Conte A (2008). The phenology and population dynamics of *Culicoides* spp. in different ecosystems in the Netherlands. Prev Vet Med.

[28] Caligiuri V, Giuliano GA, Vitale V, Chiavacci L, Travaglio S, Manelli L (2004). Bluetongue surveillance in the Campania region of Italy using a geographic information system to create risk maps. Vet Ital.

[29] Guis H, Tran A, de La Roque S, Baldet T, Gerbier G, Barraguè B (2007). Use of high spatial resolution satellite imagery to characterize landscapes at risk of bluetongue. Vet Res.

[30] Ruder MG, Howerth E, Stallknecht DE, Allison AB, Carter DL, Drolet BS, et al. Vector competence of *Culicoides sonorensis* (Diptera: Ceratopogonidae) to epizootic hemorrhagic disease virus serotype 7. Parasit Vectors. 2012;5:236. 10.1186/1756-3305-5-236 PMC3504516 23075098

